# Supramolecular polymer assembly in aqueous solution arising from cyclodextrin host–guest complexation

**DOI:** 10.3762/bjoc.12.7

**Published:** 2016-01-12

**Authors:** Jie Wang, Zhiqiang Qiu, Yiming Wang, Li Li, Xuhong Guo, Duc-Truc Pham, Stephen F Lincoln, Robert K Prud’homme

**Affiliations:** 1 State Key Laboratory of Chemical Engineering, East China University of Science and Technology, Shanghai 200237, China; 2 Departmant of Chemistry, University of Adelaide, Adelaide, SA 5005, Australia; 3 Department of Chemical Engineering, Princeton University, Princeton, NJ 08544, USA

**Keywords:** cyclodextrin, host–guest, polymer, smart-material, supramolecular

## Abstract

The employment of cyclodextrin host–guest complexation to construct supramolecular assemblies with an emphasis on polymer networks is reviewed. The main driving force for this supramolecular assembly is host–guest complexation between cyclodextrin hosts and guest groups either of which may be discrete molecular species or substituents on a polymer backbone. The effects of such complexation on properties at the molecular and macroscopic levels are discussed. It is shown that cyclodextrin complexation may be used to design functional polymer materials with tailorable properties, especially for photo-, pH-, thermo- and redox-responsiveness and self-healing.

## Introduction

Supramolecular assembly driven by associative forces including hydrogen bonding, coordinate bonding, electrostatic interactions and hydrophobic interactions is ubiquitous in nature. This is exemplified by the use of DNA and RNA complementarity [1–2] and polypeptide helix formation [3–4] to produce three-dimensional structures and materials with specific biofunctionality. Similar interactions may be utilized in the construction of functional materials. This is demonstrated in supramolecular assemblies based on cyclodextrin host–guest complexation which have attracted considerable interest through their applications in enzyme technology [5], chemical sensors [6] and drug delivery [7–9].

**Figure 1:**
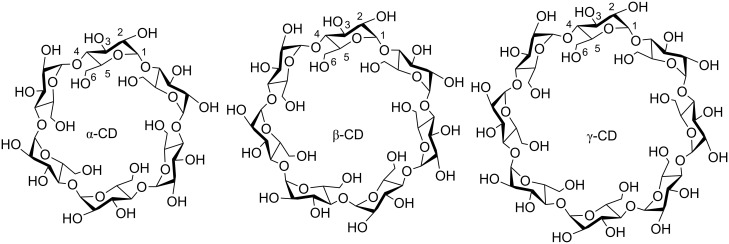
Structures of α-, β- and γ-CD. Individual carbon atom numbering is shown for one D-glucopyranose subunit in each structure.

As discussed in a range of reviews [10–14] and books [15–18], cyclodextrins are naturally occurring cyclic oligosaccharides which are also produced industrially through the enzymatic metabolism of starch and related compounds. The enzymes used are cyclodextrin glucosyltransferases which are produced by several microorganisms including *Bacillus macerans* and *Bacillus circulans*. The most common cyclodextrins are α-, β- and γ-cyclodextrin (α-, β- and γ-CD) which consist of 6, 7 and 8 α-1,4-linked D-glucopyranose subunits, respectively. Stabilized by intramolecular hydrogen bonds, cyclodextrins form truncated toroidal structures with different internal annular diameters but the same depth of 7.9 Å (Figure 1, Table 1) [19]. The primary hydroxy groups are located on the C6 carbons of the D-glucopyranose subunits and delineate the narrower, or primary, face of the torus and the secondary hydroxy groups are located on the C2 and C3 carbons and delineate the wider, or secondary, face. While the hydroxy groups on both cyclodextrin faces hydrogen bond with water in aqueous solution, the interior of the annulus is hydrophobic and selectively complexes hydrophobic guest species to form host–guest complexes, or inclusion compounds. The host–guest complexes formed by cyclodextrins and their hydrophobic guests, which range from small molecules to polymer substituents and sections of polymer chains, have been widely studied and utilized as building blocks in supramolecular structures and functional materials. These are exemplified by catenanes [20–21], rotaxanes [21–25], polyrotaxanes [24–29], polymers and polymer networks [12,22,26,30–34].

**Table 1:** Physical propertices of cyclodextrins [19].

CD	Number of D-glucopyranose subunits	Molecular weight, g/mol	Solubility in water (298.2 K), g/100 cm^3^	Narrow and wide face annular diameters, Å	Depth of annulus, Å

α-	6	972	14.5	4.7–5.3	7.9
β-	7	1135	1.85	6.0–6.5	7.9
γ-	8	1297	23.2	7.5–8.3	7.9

The focus of this review is on recent developments in the construction of supramolecular assemblies and polymer networks in water based on host–guest complexation between cyclodextrin hosts and discrete molecular entities and polymer substituents acting as guests. (Whilst the cyclodextrin torus is shown in a variety of ways in the literature, only the internal outline of the annulus is shown for uniformity and simplicity in this review.)

## Review

### 1 Host–guest complexation between cyclodextrins and guest-substituted polymers

#### 1.1 Modulation of hydrophobic interactions

Hydrophobic interactions of water soluble polymers substituted with either terminal hydrophobic substituents alone or multiple hydrophobic substituents along the polymer backbone result in aqueous solutions with tunable viscosities, diffusion characteristics and relaxation times whilst lacking undesirable thickening effects [35–36]. The extent of such hydrophobic interaction may be controlled by either the type or density of hydrophobic groups [36–37]. Alternatively, similar control may be effected through additives exemplified by a range of molecular species, salts and surfactants [38–40]. Among such additives are cyclodextrins which can disrupt the interactions between hydrophobic substituents rendering a solution viscous by forming host–guest complexes with individual hydrophobic substituents and thereby lower solution viscosity [41–44]. This process may be reversed by adding competing hydrophobes which complex cyclodextrins more strongly than the hydrophobic substituents to restore solution viscosity [45–46].

#### 1.2 Host–guest complexation of hydrophobic substituents in polymers

**Figure 2:**
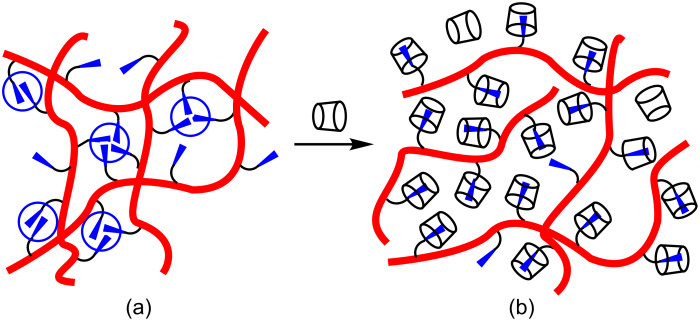
Associations of hydrophobic substituents (circled) (a) and their disruption through host–guest complexation by cyclodextrins (b).

Hydrophobic associations in aqueous solution between either terminal or multiple hydrophobic substituents along the backbone of a polymer, which generate the high viscosity of associative thickeners, may be disrupted by cyclodextrin host–guest complexation of these substituents (Figure 2) [41–49]. Thus, in 1998, Zhang et al. reported that the viscosity of an aqueous solution of perfluorocarbon-substituted poly(ethylene glycol) was decreased through the addition of β-CD due to host–guest complexation as observed by ^19^F NMR spectroscopy [47]. Subsequently, Islam et al. observed the host–guest complexation of the linear alkyl substituents *n*-C_8_H_17_, *n*-C_16_H_33_ and *n*-C_20_H_41_ of hydrophobically substituted alkali-soluble emulsion (HASE) polymers by methylated β-CD using gel permeation chromatography and light scattering methods [48]. In 2002, Karlson et al. found that hydrophobic association among the hydrophobic substituents of substituted poly(ethylene glycol) was disrupted by host–guest complexation by methylated α-CD [42]; as was a similar association by the hydrophobic substituents of substituted ethyl(hydroxyethyl) cellulose by α-CD, β-CD and their methylated analogs [49].

**Figure 3:**
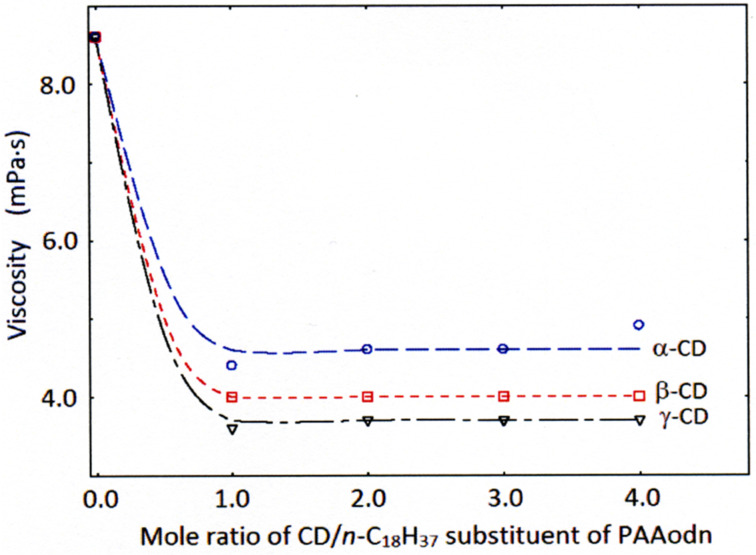
Decrease of aqueous solution viscosity at a shear rate of 50 s^−1^ due to α-CD (circles), β-CD (rectangles) and γ-CD (triangles) host–guest complexation of *n*-C_18_H_37_ substituents competing with *n*-C_18_H_37_ hydrophobic interactions in the randomly substituted poly(acrylate), PAAodn, 0.5 wt % aqueous solution (0.10 M NaCl, pH 7.0). Adapted with permission from [46]. Copyright (2008) American Chemical Society.

Guo et al. have shown that the viscosity of aqueous solutions 0.5 wt % in 2% *n*-C_12_H_25_, *n-*C_14_H_29_ or *n*-C_18_H_37_ randomly substituted poly(acrylate) (PAAddn, PAAtdn and PAAodn, respectively) is significantly decreased upon addition of α-, β- or γ-CD due to decreased hydrophobic interaction between the *n*-alkyl substituents because of their cyclodextrin host–guest complexation [46]. Due to the differences in annular size (Table 1), the hydrophobe complexing abilities of α-, β- and γ-CD differ [44–46]. At low PAAodn 0.5 wt % concentration in aqueous solution, the viscosity decreases substantially to a minimum value at either 1:1 α-CD, β-CD or γ-CD host to *n*-C_18_H_37_ guest substituent mole ratio (Figure 3) [46]. This minimum viscosity value decreases on going from α-CD to γ-CD due to the stronger complexation of a single *n*-C_18_H_37_ substituent with increasing size of the cyclodextrin annulus.

**Figure 4:**
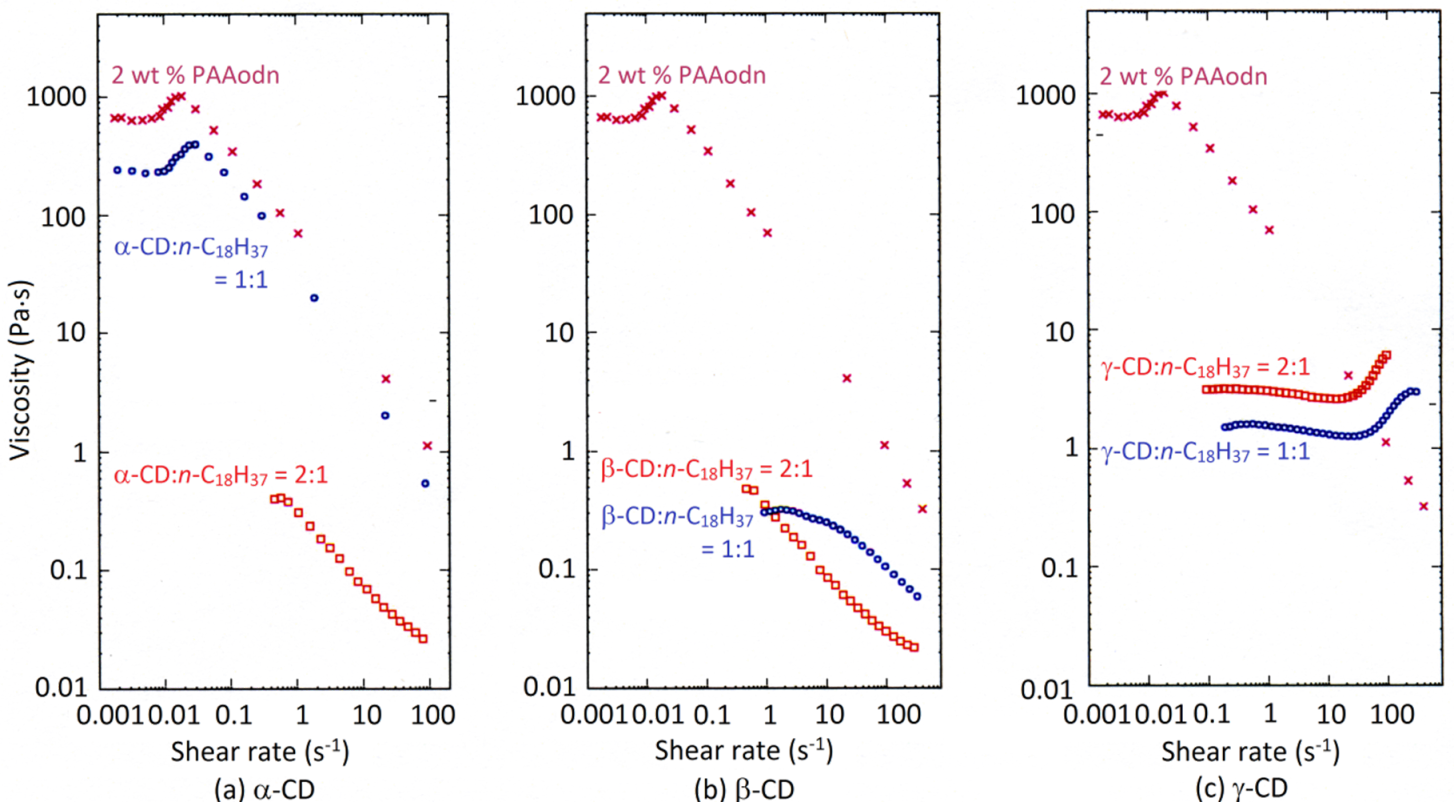
The effect of (a) α-CD, (b) β-CD and (c) γ-CD on the hydrophobic interactions between *n*-C_18_H_37_ substituents of 2% randomly substituted poly(acrylate), PAAodn, in 2 wt % aqueous solution (0.10 M NaCl, pH 7.0) as indicated by shear rate. The data sets refer to 2 wt % PAAodn alone (crosses), and where cyclodextrin to *n*-C_18_H_37_ substituent mole ratios are: 1:1 (circles) and 2:1 (rectangles). Adapted with permission from [46]. Copyright (2008) American Chemical Society.

At a higher PAAodn concentration (2 wt %), the viscosity behavior changes with the increase in the cyclodextrin mole ratio (Figure 4) [46]. Thus, at a 1:1 α-CD:*n*-C_18_H_37_ mole ratio the solution viscosity decreases by almost a half and the viscosity profile is little changed (Figure 4a). This is consistent with *n*-C_18_H_37_ partially protruding from the narrow α-CD annulus such that residual hydrophobic interactions occur between *n*-C_18_H_37_ substituents and substantial viscosity is retained. However, when the α-CD:*n*-C_18_H_37_ mole ratio increases to 2:1 the viscosity decreases by almost three orders of magnitude and further addition of α-CD has little effect. This is consistent with a 2:1 2α-CD:*n*-C_18_H_37_ host guest stoichiometry being assumed where two α-CD thread onto a single *n*-C_18_H_37_ substituent such that interaction between substituents decreases greatly. In contrast, at 1:1 β-CD:*n*-C_18_H_37_ mole ratio the solution viscosity decreases greatly and further addition of β-CD has only a small effect (Figure 4b). This is consistent with a β-CD:*n*-C_18_H_37_ host–guest stoichiometry dominating and *n*-C_18_H_37_ folding inside the larger β-CD annulus such that little residual interaction between the *n*-C_18_H_37_ hydrophobic substituents occurs. Nevertheless, the expected shear thickening occurs with increasing shear rate in the presence of both α-CD and β-CD. The effect of addition of γ-CD is quite different and probably reflects the effect of a 1:1 γ·CD:*n*-C_18_H_37_ host–guest stoichiometry dominating at low to moderate shear rates (Figure 4c). At higher shear rates, a γ-CD/2*n*-C_18_H_37_ host–guest stoichiometry in which the large γ-CD annulus accommodates two *n*-C_18_H_37_ from adjacent PAAodn chains becomes increasingly significant and shear thickening occurs.

#### 1.3 Recovery of hydrophobic association

**Figure 5:**
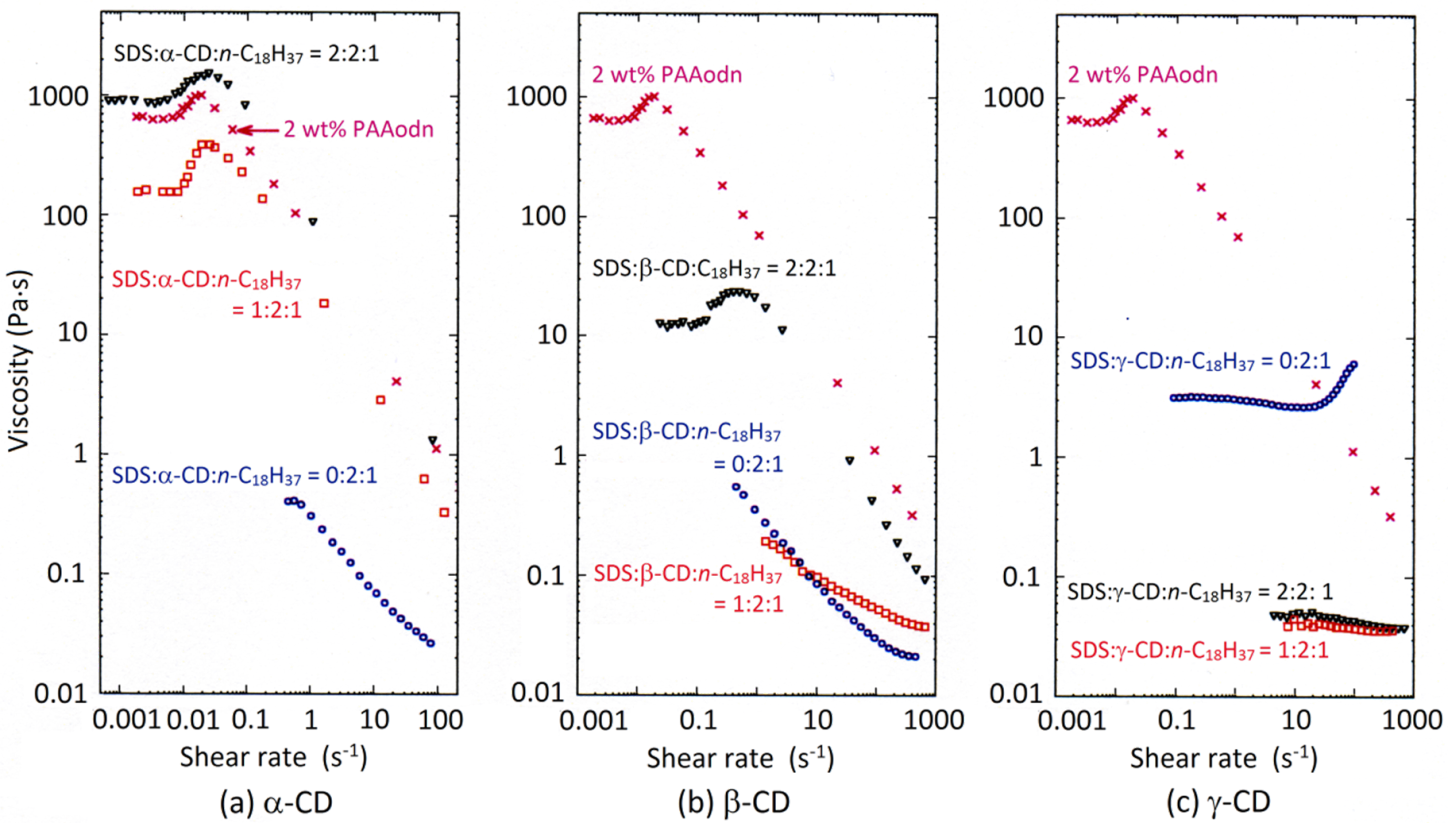
The effect of SDS addition on viscosity shear rate dependence for 2 wt % aqueous PAAodn solutions containing (a) α-CD, (b) β-CD and (c) γ-CD with a molar ratio of CD to *n*-C_18_H_37_ = 2:1 (0.10 M NaCl, pH 7.0). Data sets are shown for 2 wt % PAAodn alone (crosses) and for the ratios: SDS:CD:*n*-C_18_H_37_ = 0:2:1 (circles), SDS:CD:*n*-C_18_H_37_ = 1:2:1 (rectangles) and SDS:CD:*n*-C_18_H_37_ = 2:2:1 (triangles). Adapted with permission from [46]. Copyright (2008) American Chemical Society.

Hydrophobic associations in substituted polymer solutions may be recovered by adding other guest species which form more stable cyclodextrin host–guest complexes than the polymer substituents do [41,45–46,50]. Thus, Khan et al. used nonionic surfactants based on poly(ethylene glycol) to recover the hydrophobic associations in hydrophobically substituted alkali-soluble emulsion (HASE) polymers complexed by α-CD and β-CD [41]. (It should be noted that association occurs between hydrophobically substituted polymers in aqueous solution and that this may be decreased by the addition of surfactants as shown by Prud’homme et al. for hydrophobically substituted hydroxyethyl cellulose [51].) Guo et al. showed that the addition of sodium dodecyl sulfate (SDS) to 2 wt % aqueous PAAodn in which the α-CD:*n-*C_18_H_37_ mole ratio is 2:1 to make the mole ratios of SDS:α-CD:*n-*C_18_H_37_ 1:2:1 and 2:2:1 causes viscosity to closely approach and to exceed that of 2 wt % aqueous PAAodn, respectively (Figure 5a) [45–46]. Further addition of SDS causes solution viscosity to decrease. This is consistent with α-CD complexing SDS more strongly than *n-*C_18_H_37_ such that hydrophobic interactions between PAAodn are restored in the 1:2:1 and 2:2:1 solutions while at higher SDS ratios SDS dominated micelles form which disrupt inter-polymer chain interactions [37,40,51]. Similar additions of SDS to the 2:1 β-CD:*n-*C_18_H_37_ solution restores the hydrophobic interactions between PAAodn and viscosity but to a lesser extent than for the 2:1 α-CD:*n-*C_18_H_37_ solution consistent with the *n-*C_18_H_37_ substituents competing more effectively with SDS in host–guest complexation with β-CD (Figure 5b). Addition of SDS decreases viscosity and removes the shear thickening observed for the 2:1 γ-CD:*n-*C_18_H_37_ solution probably as a result of the larger γ-CD simultaneously complexing both *n-*C_18_H_37_ and SDS such that complexation of two *n*-C_18_H_37_ by γ-CD is minimized (Figure 5c).

### 2 Network assembly by cyclodextrin- and guest-substituted polymers

The ability of cyclodextrins to complex hydrophobic guests in aqueous solution may be used to greatly extend supramolecular and polymer chemistry when cyclodextrins and hydrophobes are substituted onto water-soluble polymer backbones. The host–guest complexes formed between the cyclodextrin and hydrophobic substituents represent very specific interactions between polymer chains which may be exploited to modulate the polymer networks formed and the viscosities of their aqueous solutions.

#### 2.1 Construction of polymer networks

**Figure 6:**
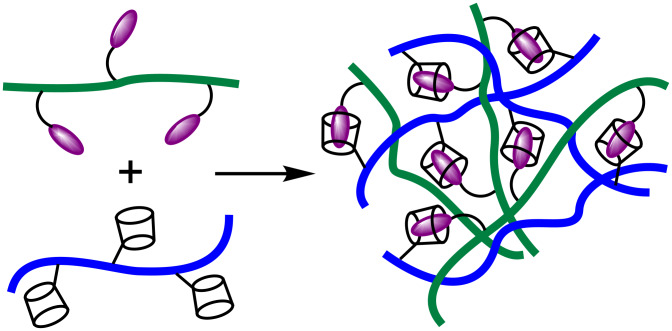
Host–guest complexation between polymers with cyclodextrin and hydrophobic substituents.

The formation of a polymer network through host–guest complexation between cyclodextrin and hydrophobic substituents on different polymer chains is illustrated in a general manner in Figure 6. Such network formation is exemplified by the research of Wenz et al. in which mixtures of poly(maleic acid)-*co*-(isobutene) copolymers substituted with either β-CD or 4-*tert*-butylanilide form viscous aqueous solutions as host–guest complexation between these substituents form a polymer network [52–53]. Gosselet et al. [54–55] and Cammas et al. [56], respectively, mixed the adamantyl-substituted *N*,*N*-dimethylacrylamide hydroxyethylmethacrylate and β-malic acid-co-ethyladamantyl β-malate copolymers with β-CD-substituted-epichlorohydrin copolymers to obtain highly viscous solutions as a result of polymer network formation occurring through host–guest complexation between the β-CD and adamantyl substituents of the polymers.

**Figure 7:**
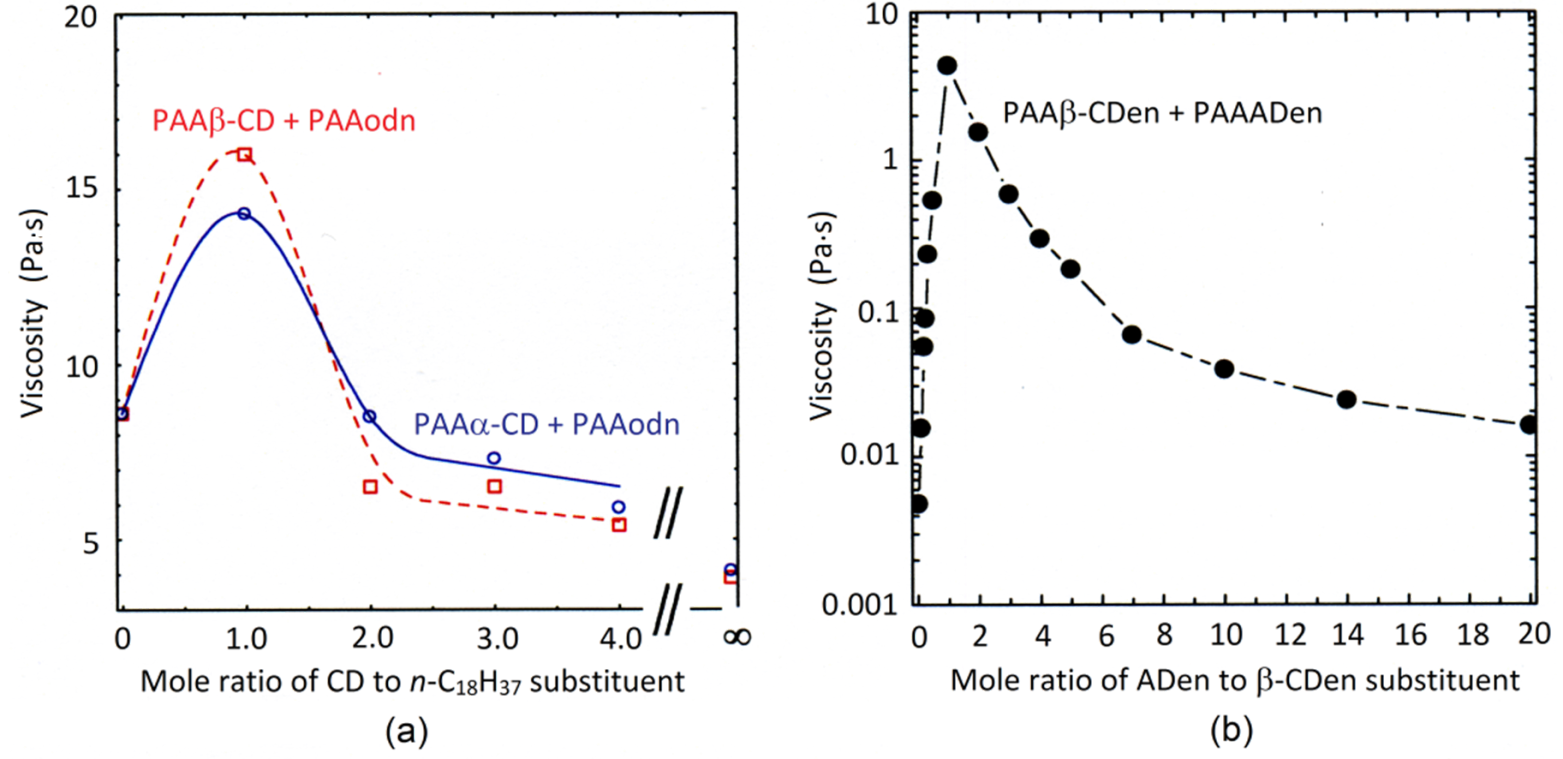
Variation of viscosity with mole ratio of CD substituents to hydrophobic substituents on poly(acrylate), PAA. (a) 0.5 wt % aqueous solutions of, respectively, 2.5 and 2.1% α-CD and β-CD randomly substituted PAA (PAAα-CD and PAAβ-CD) and *n*-C_18_H_37_ 3% randomly substituted PAA (PAAodn) [44]. Adapted with permission from [44]. Copyright (2005) American Chemical Society. (b) 2.0 wt % aqueous solution of 2.9% β-CDen randomly substituted PAA (PAAβ-CDen) and 3.0% ADen randomly substituted PAA (PAAADen) [57]. Adapted with permission from [57]. Copyright (2008) American Chemical Society.

Guo et al. prepared substituted poly(acrylate) networks through host–guest complexation between either the α-CD or β-CD substituents of PAAα-CD and PAAβ-CD and the *n*-C_18_H_37_ substituents of PAAodn [44], and also the 1-(2-aminoethyl)amido-β-CD (β-CDen) and 1-(2-aminoethyl)amidoadmantyl (ADen) substituents on the substituted poly(acrylate)s PAAβ-CDen and PAAADen, respectively [57]. The host–guest complexations between the cyclodextrin substituents and both *n*-C_18_H_37_ and ADen substituents in PAAodn and PAAADen, respectively, have a 1:1 stoichiometry. In both cases, the solution viscosity reaches a maximum when the host:guest substituent ratio is 1:1 and decreases when one substituent concentration exceeds the other as the substituted poly(acrylate) in excess concentration decreases the overall participation in network formation and thereby lowers solution viscosity (Figure 7a and b).

In principle these are good model systems to quantitatively test theories of polymer association exemplified by the studies of Tanaka and Edwards [58] and Rubinstein et al. [59–62]. However, matching experiment to theory remains a considerable challenge as associative polymer networks can incorporate clusters each containing 10–30 hydrophobic substituents depending on the polymer concentration as shown by the fluorescence studies of Winnik et al. [63–65].

#### 2.2 Comparison of guests

Cyclodextrin host–guest complexation of guest species in aqueous solutions is largely driven by van der Waals and hydrophobic interactions between the interior of the cyclodextrin annulus and the guest with dehydration of both substantially influencing the thermodynamics of the process [66]. As a result, the guest often exhibits a substantial change in its UV–vis, fluorescence and ^1^H NMR spectra upon complexation, and there is usually a significant enthalpy change. Consequently, UV–vis [67], fluorescence [68] and ^1^H NMR [69] spectroscopy and isothermal titrimetry calorimetry, ITC [52,70], are frequently used in characterizing host–guest complexation. When viscosity changes occur because of host–guest complexation, rheology may be used to characterize such complexation [71]. Some examples of aqueous polymer systems characterized by these techniques appear in Table 2.

**Table 2:** Host–guest complexation systems, complexation constants and methodologies.

Polymer backbone	Guest substituent	Host	*K* (M^−1^)	Method

poly(acrylate)	azobenzene	3α-CD^a^	140	UV–vis [67]
poly(acrylate)	azobenzene	6α-CD^a^	12000	UV–vis [67]
poly(acrylamide)	(1-naphthyl)methyl	6β-CD^a^	77	fluorescence [68]
poly(acrylamide)	(2-naphthyl)methyl	6β-CD^a^	190	fluorescence [68]
poly(methacrylamide)	tryptophan	α-CD^b^	30	^1^H NMR [69]
poly(methacrylamide)	tryptophan	β-CD^b^	83	^1^H NMR [69]
poly(methacrylamide)	tryptophan	γ-CD^b^	11	^1^H NMR [69]
poly(maleate)-*co*-(isobutene)	4-*tert*-butylphenyl	3β-CD^a^	25900	ITC [52]
poly(acrylate)	adamantyl	6β-CD^a^	3020	ITC [70]
HASE polymer	C_22_H_45_	α-CD^b^	11100	rheology [71]
HASE polymer	C_22_H_45_	β-CD^b^	1890	rheology [71]

^a^The 3α-CD, 6α-CD and 6β-CD substituents are tethered to the polymer backbone through the 3C carbon of a single D-glucopyranose subunit in the first case, and through the C6 carbon in the second and third cases. ^b^Free cyclodextrin.

#### 2.3 Effect of substituent tether length in substituted polymers

**Figure 8:**
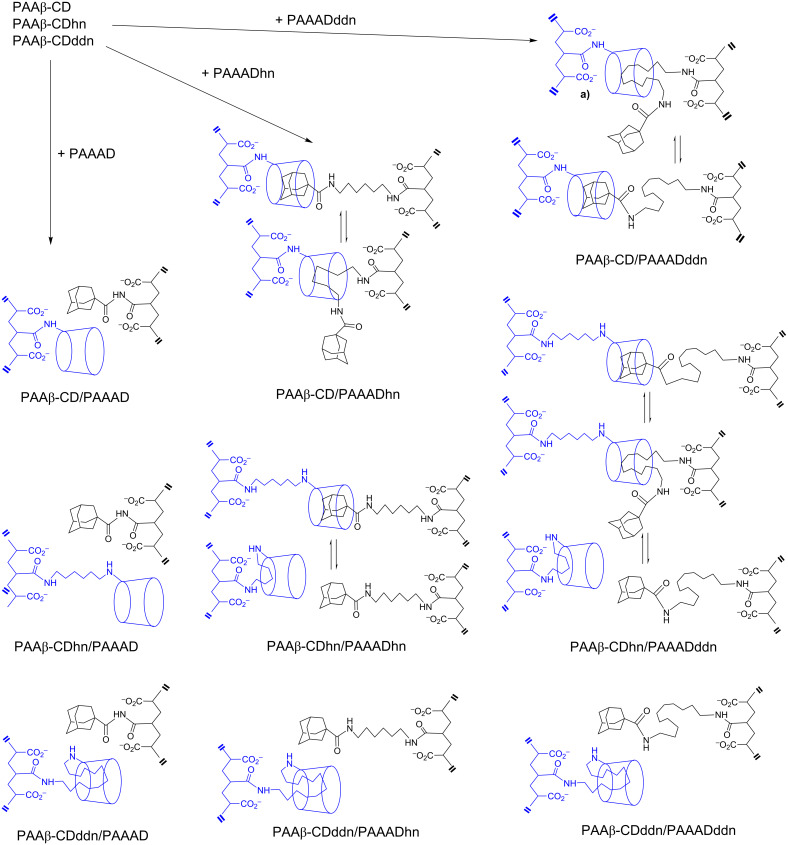
Illustration of the competitive intermolecular host–guest complexation of either the adamantyl substituent or the tethers of the adamantyl and β-CD substituents in a range of substituted poly(acrylate) systems. In each case β-CD is tethered to the PAA backbone through a C6 carbon in a D-glucopyranose subunit of each β-CD. Reproduced with permission from [72]. Copyright (2010) Wiley-VCH.

Host–guest complexation in substituted polymer systems is substantially affected by the length of the tether through which either the cyclodextrin or hydrophobe is attached to the polymer backbone. This also affects the extent of intramolecular interactions between substituents in a single polymer chain and of intermolecular interactions between substituents in adjacent polymer chains. Consequently, the strength of interaction between the substituted poly(acrylates)s is substantially controlled by the variation in occurrence of intra- and inter-molecular host–guest complexation between the β-CD substituents tethered by amido, diacylamino-1,6-hexyl and diacylamino-1,12-dodecyl tethers in the respective substituted poly(acrylate)s, PAAβ-CD, PAAβ-CDhn and PAAβ-CDddn, and the similarly tethered adamantyl (AD) substituents in the PAAAD, PAAADhn and PAAADddn substituted poly(acrylate)s shown in Figure 8 [72]. The substituent tether length largely controls the relative importance of the intra- and inter-molecular complexation modes and also the extent to which the adamantyl substituent and its tether and the β-CD tether compete for host–guest complexation in the β-CD substituent annulus to form interchain linkages in the polymer network as shown by 2D ^1^H NMR spectroscopy. Rheological studies show that as its length shortens the tether is less able to compete for β-CD substituent annular occupancy, and that the coincident increase in steric interactions with the poly(acrylate) backbone also inhibits intermolecular host–guest complexation [72–73].

### 3 Polymer network assembly through covalently-linked cyclodextrins

**Figure 9:**
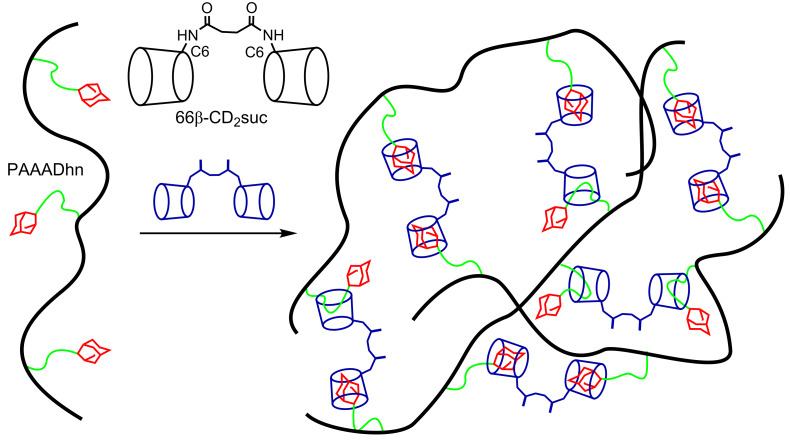
Competitive host–guest complexations in which either the adamantyl substituent (red) or the *n*-hexyl tether (green) of PAAADhn is the guest in the 66β-CD_2_suc annuli to form a hydrogel network [76].

The simplest covalently-linked cyclodextrins are dimers which may act as ditopic hosts due to the presence of the two cyclodextrin annuli. Thus, such dimers may be used to form cross-links through the complexation of hydrophobic substituents on adjacent polymer chains and thereby generate a polymer network and hydrogel [74–75]. Variation of the length of the covalent-linker in the cyclodextrin dimer and of the tether between the hydrophobic substituents and the polymer backbone can substantially affect the host–guest interactions as is illustrated by studies of β-CD dimers and adamantyl-substituted poly(acrylate)s (Figure 9) [76]. (A similar situation also prevails for covalently-linked β-CD trimers as shown by Lincoln et al. [77].) The longer succinamide linker in 66β-CD_2_su engenders higher viscosities than does the shorter urea linker in 66β-CD_2_ur probably because steric hindrance between the adjacent adamantyl-substituted poly(acrylate) chains is greater when 66β-CD_2_ur forms a cross-link [76]. (The 66 prefix in 66β-CD_2_su and 66β-CD_2_ur indicates that the succinamide and urea linkers are attached to the C6 carbon in a D-glucopyranose subunit of each β-CD.) The increasing length of the adamantyl tether from amido to hexylamido in PAAAD and PAAADhn progressively decreases steric hindrance between the poly(acrylate) backbones and facilitates host–guest complexation such that polymer network formation strengthens. Competition between the adamantyl group and its hexyl tether for complexation in the annuli of 66β-CD_2_su also occurs (Figure 9). Interestingly, as length increases further to twelve methylene groups in the dodecyl tether in PAAADddn, a particularly marked decrease in the viscosity of the hydrogel formed with 66β-CD_2_ur occurs by comparison with that formed with PAAADhn. This may be partly attributed to the increased flexibility allowed by the longer tether in the polymer network formed when host–guest complexation occurs.

**Figure 10:**
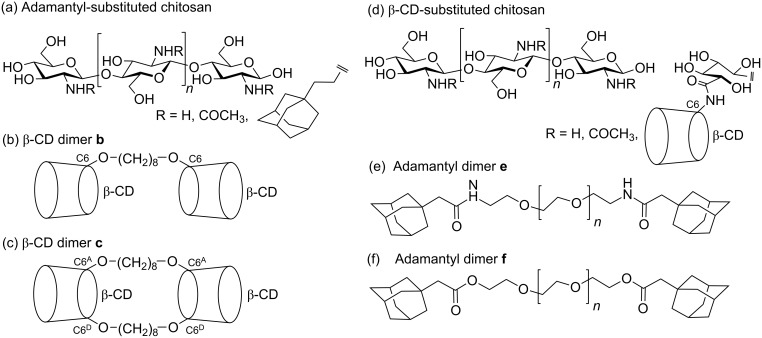
(a) Substituted chitosan in which acyl- and adamantyl-substitution is 5% and 12 %, respectively. (b) Octylmethylene-linked β-CD dimer **b** where substitution is at C6 for each β-CD. (c) Octymethylene-linked β-CD dimer **c** where substitution is at C6^A^ and C6^D^ for each β-CD [78] (d) Substituted chitosan in which acyl- and β-CD-substitution is 12% and 10%, respectively. (e) Diaminopolyethyleneglycol-linked adamantyl dimer **e**. (f) Polyethyleneglycol-linked adamantyl dimer **f** [81].

In earlier studies, Auzély-Vetly et al. reported the substitution of chitosan (molecular weight 195 kDa) with adamantyl groups and characterized their complexation in the β-CD annuli of a singly octamethylene-linked β-CD dimer **b** and a doubly octamethylene-linked β-CD dimer **c** (Figure 10a, b and c) [78] and related β-CD [79–80] dimers in aqueous solution. It was determined from ITC experiments that while β-CD formed a 1:1 host–guest complex with adamantane carboxylate, only one annulus of the β-CD dimer **b** and β-CD dimer **c** complexed adamantane carboxylate on average. This was attributed to aggregation of the dimers as a consequence of their amphiphilic nature, complexation of the octamethylene linker in the β-CD dimer annuli, and hydrogen bonding interactions between their β-CD annuli. The 1:1 complexation constants, 10^−4^*K*_11_ = 7.96, 2.32 and 26.42 M^−1^ in aqueous solution at 298.2 K for β-CD and the β-CD dimer **b** and the β-CD dimer **c**, respectively, where the greater β-CD dimer **c** complex stability was attributed to the greater hydrophobicity arising from the two octamethylene linkers. Rheological studies of aqueous solutions of adamantyl-substituted chitosan showed a moderate increase in viscosity with increase in β-CD dimer **b** concentration at a constant substituted-chitosan concentration consistent with the formation of cross-links forming through ditopic complexation by the β-CD dimer **b** of adamantyl substituents on adjacent chitosan chains. A much greater increase in viscosity was observed when the β-CD dimer **c** was employed consistent with its greater rigidity derived from the twin octamethylene linkers enhancing interchain cross-link formation.

Interesting variations on the above complexation studies are those relating to β-CD-substituted chitosan and the diamino-poly(ethylene glycol)-linked adamantyl dimer **e** and the poly(ethylene glycol)-linked adamantyl dimer **f**, in which the linker molecular weight is either 3.4 or 20 kDa in each case, shown in Figure 10d, e and f, respectively [81]. Rheological studies of aqueous solutions of β-CD-substituted chitosan show increased viscosity in the presence of adamantyl dimers **e** and **f** consistent with the formation of cross-links forming through complexation of the adamantyl groups of the dimers by β-CD substituents on adjacent chitosan chains.

### 4 Threading cyclodextrins onto polymer backbones

Since the report of host–guest complexation between α-CD and poly(ethylene glycol) (PEG) by Harada and Kamachi in 1990 [82], a variety of pseudo-polyrotaxanes and polyrotaxanes formed through host–guest complexation between cyclodextrins and linear polymers have been reported [83], some of which form hydrogels [84–85]. In particular, hydrogels formed by PEG and cyclodextrins have been investigated intensively because of the biocompatibility of their components. Interestingly, local crystallization of the polyrotaxane threaded cyclodextrins, sometimes called molecular necklaces [86], may form cross-links and polymer networks in aqueous solution. In 1994, Li et al. reported the formation of hydrogels based on the host–guest complexation between α-CD and high molecular weight PEG [87]. They found the hydrogel melting temperature to increase with increase in PEG length and α-CD concentration and to decrease with increase in PEG concentration consistent with the threading of varying numbers of α-CD onto the PEG. It was also observed that X-ray powder diffraction patterns of the powdered frozen hydrogel were consistent with the formation of localized regions where the α-CD/PEG pseudo-polyrotaxanes crystallized to form interchain links within the hydrogel. Similar conclusions were reached from another X-ray powder diffraction study of frozen α-CD/PEG hydrogels formed with PEG of 8, 20 and 600 kDa molecular weights [87]. The accompanying rheological and differential scanning calorimetric studies were also consistent with the localized crystallization of α-CD/PEG pseudo-polyrotaxanes forming interchain cross-links in the hydrogel.

**Figure 11:**
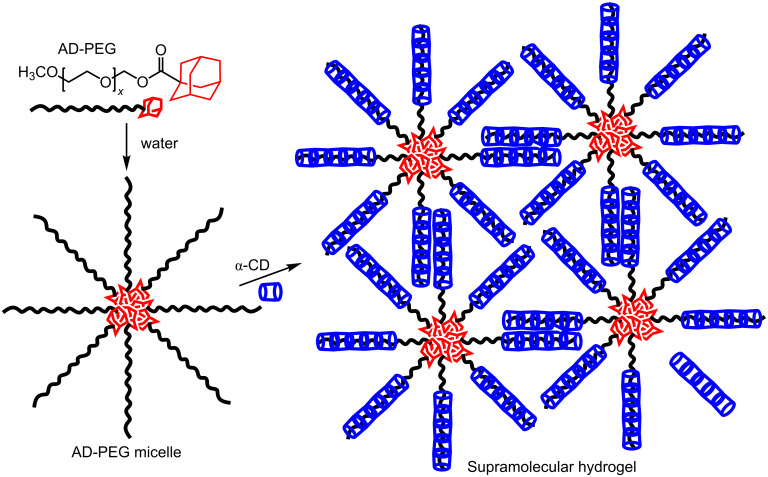
The formation of a AD-PEG micelle followed by the formation of a AD-PEG/α-CD supramolecular hydrogel in aqueous solution [88].

Low molecular weight <2 kDa PEG forms crystalline precipitates in aqueous solutions in the presence of α-CD largely because multiple α-CD thread onto the PEG chain such that the uncomplexed portions of the PEG chains are too short for significant interchain interaction to form a water soluble network [82,86,88]. However, when a hydrophobic adamantyl group is substituted onto one end of a low molecular weight PEG chain to form amphiphilic AD-PEG, it is found that the adamantyl substituents aggregate in aqueous solution to form a micelle and that subsequent addition of α-CD leads to the formation of a supramolecular hydrogel as shown in Figure 11 [88]. The driving force for gelation is a combination of the hydrophobic aggregation of the adamantyl substituents and the aggregation of the α-CD complexed portions of the AD-PEG chains. Part of the interest in these low molecular weight polymer systems arises because they are able to pass through the kidney membrane [89] and are consequently of interest as components of drug-delivery systems [89–90].

**Figure 12:**
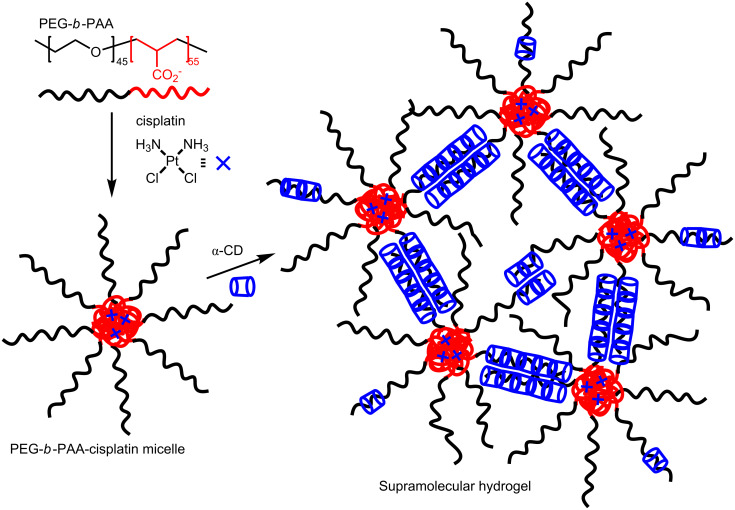
Interaction of PEG-*b*-PAA block copolymer with *cis*-diamminedichloroplatinum(II), cisplatin, to form a PEG-*b*-PAA-cisplatin micelle followed by the addition of α-CD to form a supramolecular hydrogel [92].

A hydrogel formed through the initial formation of micelles of poly(ethylene glycol)-*b*-poly(acrylate), PEG-*b*-PAA, copolymer and the widely used anticancer drug cis-diamminedichloroplatinum(II), cisplatin [91], and subsequent host–guest complexation by α-CD has been developed by Zhu et al. (Figure 12) [92]. In the first stage, the two chloro ligands on the four-coordinate square-planar platinum(II) center of cisplatin are displaced by PEG-*b*-PAA carboxylate groups to produce PEG-*b*-PAA-cisplatin micelles. Addition of α-CD results in host–guest complexation of the PEG segments of PEG-*b*-PAA and subsequent aggregation of these α-CD-complexed PEG molecular necklace segments to form a network and a supramolecular hydrogel. Because the interactions between the α-CD-complexed PEG segments forming cross-links are non-covalent and quite weak, their aggregations can be broken by applying shear force such that the reversibility of the solution/hydrogel transition is observed in rheological experiments. In vitro tests show that the PEG-*b*-PAA/cisplatin hydrogel has a sustained cisplatin release over three days and that it has a high cytotoxity towards human bladder carcinoma EJ cells.

### 5 Responsive smart materials

Cyclodextrin host–guest chemistry is characterized by an ever-expanding range of host and guests, some of which undergo structural and interaction variations in response to stimuli as exemplified by photo-, pH-, temperature- and redox-responsive changes. Consequently, cyclodextrins have generated a range of stimuli-responsive materials collectively called “smart materials”. These include “self-healing materials” which after being disrupted can recover their former state through host–guest interactions. Some examples of these materials are now discussed.

#### 5.1 Photo-responsive materials

**Figure 13:**
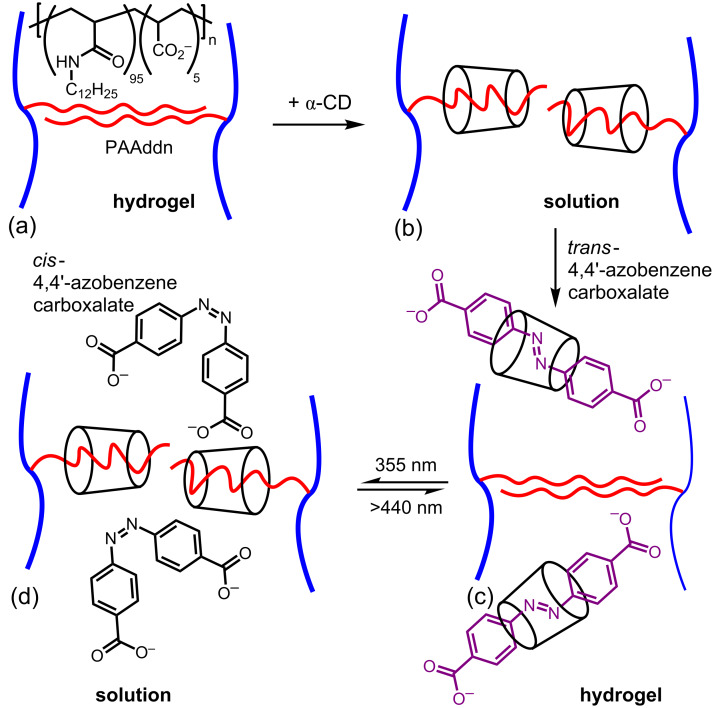
Solution to hydrogel transitions (a)–(d) for a PAAddn segment in the presence of competitive photo-responsive complexation of the dodecyl substituent by α-CD and *E*- or *trans*-4,4’-azobenzene carboxylate and its *Z*- or *cis*-isomer in basic aqueous solution [93].

Among the better known photo-responsive small molecules are azobenzene and its derivatives which isomerize from *trans* to *cis* and from *cis* to *trans* under irradiation with UV and visible light, respectively, and are potentially components of photo-responsive materials. In 2005, Harada et al. constructed a photo-responsive dodecyl substituted poly(acrylate), PAAddn, hydrogel system which depends on α-CD complexing *trans*-4,4’-azodibenzoic acid but not its *cis* isomer as seen in Figure 13 [93]. Thus, alone PAAddn forms a hydrogel due to the hydrophobic interchain interactions of its dodecyl substituents. However, upon addition of α-CD the dodecyl substituents are complexed and the hydrogel is disrupted to give a free-flowing solution. The addition of *trans*-4,4’-azobenzene carboxylate to this solution results in the preferential formation of the α-CD·*trans*-4,4’-azobenzene carboxylate host–guest complex and the PAAddn hydrogel reforms. Irradiation at 335 nm causes *trans*-4,4’-azobenzene carboxylate to photo-isomerize to the *cis* isomer which is too sterically hindered to form a stable α-CD complex, and the dodecyl substituents of PAAddn are once again complexed by α-CD and the hydrogel disaggregates. This last step is reversible though irradiation at >440 nm such that the equilibria may be switched to and fro by irradiating at 350 nm, when the viscosity rises to ~3 × 10^3^ Pa·s, and >440 nm when the viscosity decreases to ~2 × 10^−2^ Pa·s.

**Figure 14:**
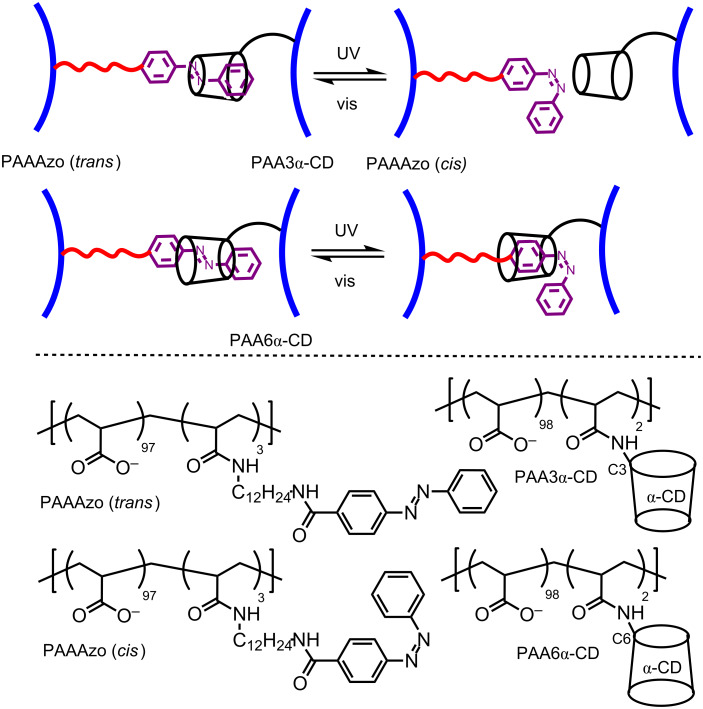
Structures of the poly(acrylate)-based polymers PAAAzo (*trans*), PAAAzo (*cis*), PAA3α-CD and PAA6α-CD, and the effects of the stereochemistry and photo-isomerism of the diazo substituents of PAA3β-CD and PAA6β-CD on their host–guest complexation by the α-CD substituents of PAA3α-CD and PAA6α-CD [67].

**Figure 15:**
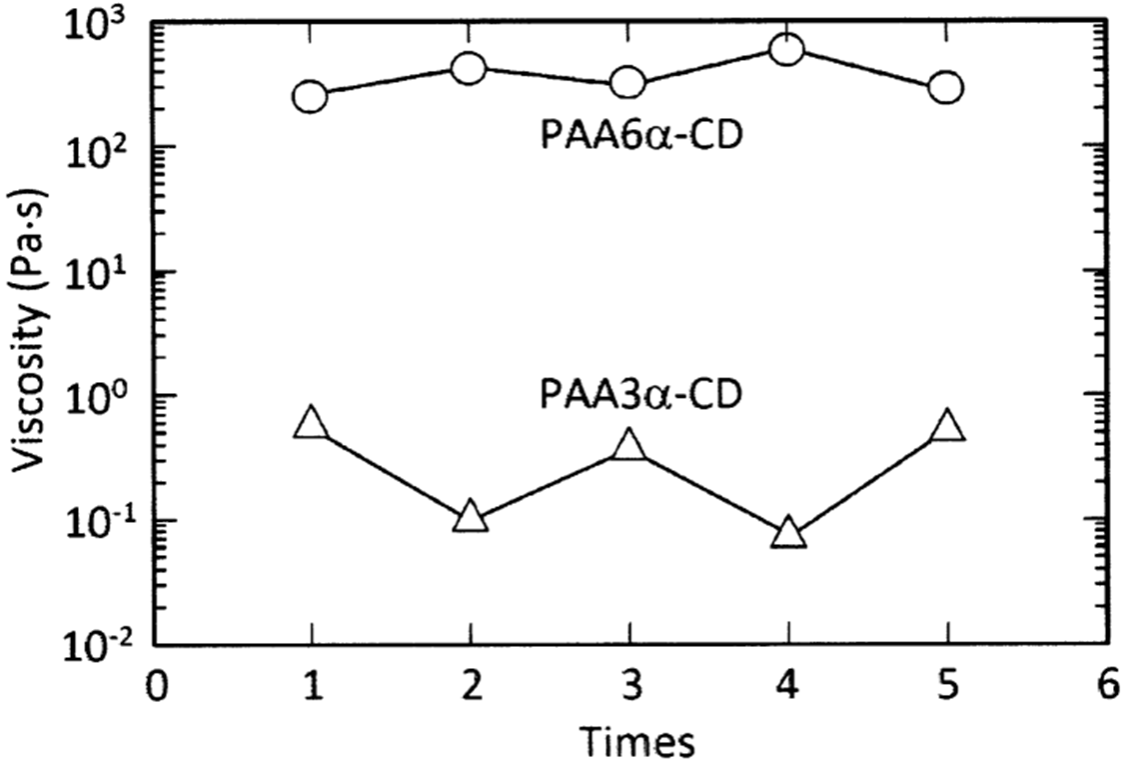
Variation of viscosity of a PAA6α-CD/PAAAzo solution (circles) and a PAA3α-CD/PAAAzo solution (triangles) during repetitive irradiations. For PAA3α-CD/PAAAzo the low and high viscosity values are attained after UV and visible irradiation, respectively, and vice versa for PAA6α-CD/PAAAzo. Adapted with permission from [67]. Copyright (2006) American Chemical Society.

Harada et al. also constructed two other photo-responsive hydrogels from a 2.7% azobenzene-substituted poly(acrylate), PAAAzo, and two α-CD-substituted poly(acrylates) in which α-CD is substituted onto the poly(acrylate) backbone through either the C3- or C6-carbon of a D-glucopyranose subunit, PAA3α-CD and PAA6α-CD, respectively, which are 1.6 and 2.2% substituted (Figure 14) [67]. The PAA3α-CD/PAAAzo and PAA6α-CD/PAAAzo host–guest complexation between the α-CD and azobenzene substituents are characterized by complexation constants, *K* = 1.4 × 10^2^ and 1.2 × 10^4^ M^−1^, respectively. Under visible light the viscosities of PAA3α-CD/PAAAzo and PAA6α-CD/PAAAzo are 6.5 × 10^−1^ and 2.5 × 10^2^ Pa·s at 298.2 K, respectively, and upon ultraviolet radiation these values decrease ten-fold and increase two-fold reversibly, respectively (Figure 15). This reflects the lesser ability of the 3α-CD substituent to complex either the *trans*- or *cis*-azobenzene substituent by comparison with the 6α-CD substituent because of the difference in steric constraint caused by the tether attachment at either the C3 or C6-carbon of a D-glucopyranose subunit, respectively.

A study by Hu et al. of azobenzene-substituted hydroxypropyl methylcellulose (azo-HPMC) polymers shows that the azobenzene substituents are reversibly photo-isomerized from the *trans* to *cis* configurations and vice-versa by ultra-violet and visible radiation, respectively, and that the corresponding solution to hydrogel transition temperatures are 299.7 K and 309.7 K for 10 g L^−1^ azo-HMPC in aqueous 0.5 M NaCl [94]. This is attributed to the *trans*-azobenzene substituents undergoing hydrophobic stacking more effectively than the less planar and more polar *cis*-azobenzene substituents. However, when 3 × 10^−2^ M^−1^ α-CD is present the solution to hydrogel transition temperatures increase to 330.2 K and 322.2 K for azo-HMPC bearing *trans*- and *cis*-azobenzene substituents, respectively. Host–guest complexation by α-CD eliminates the possibility of hydrophobic stacking between the *trans*-azobenzene substituents, but the *cis*-azobenzene substituents are not significantly complexed by α-CD and can undergo some hydrophobic stacking. In addition the relatively hydrophilic exterior of α-CD minimizes any hydrophobic attraction between the α-CD-complexed *trans*-azobenzene substituents. The solution to hydrogel transition temperature is also dependent on the extent of azobenzene substitution of azo-HMPC and the concentration of α-CD.

Another notable example of a photo-responsive hydrogel activated by the photo-isomerism of azobenzene substituents has been provided by Zhao and Stoddart [95]. In this case the *trans* azobenzene substituents of a substituted poly(acrylate) are complexed by β-CD substituted at the C3 carbon of a D-glucopyranose subunit with deoxycholic acid, and hydrophobic association between them form interchain cross-links and a hydrogel. Upon irradiation at 355 nm, *trans* to *cis* photo-isomerization of the azobenzene substituents occurs, its β-CD complexation dissociates, the deoxycholic acid moieties complex within the β-CD annuli and the hydrogel network disassociates. This process is reversible through irradiation at 450 nm.

#### 5.2. pH-Responsive materials

**Figure 16:**
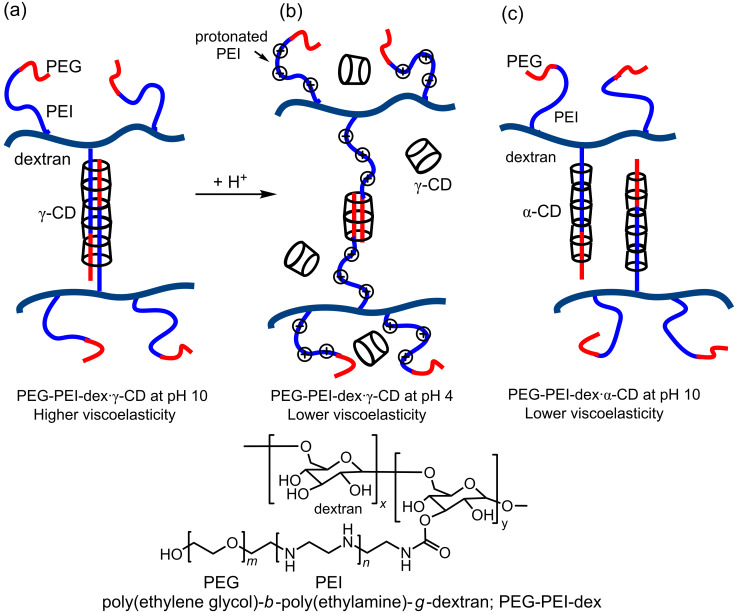
The structures proposed for the poly(ethylene glycol)-*b*-poly(ethylamine)-*g*-dextran·γ-CD, PEG-PEI-dex·γ-CD, supramolecular hydrogel at (a) pH 10 and (b) its much lower viscoelastic protonated form at pH 4. The proposed structure of the PEG-PEI-dex·α-CD at pH 10 is shown in (c) [96].

In 2007, Yui et al. reported a pH-responsive polymer system in which the simultaneous host–guest complexation by γ-CD of two of the 3.4 kDa average molecular weight poly(ethylene glycol)-*b*-poly(ethylamine) strands substituted onto the dextran backbone of a poly(ethylene glycol)-*b*-poly(ethylamine)-*g*-dextran copolymer, PEG-PEI-dex, is considered to form the network underlying the supramolecular hydrogel formed in aqueous solution at pH 10 as shown in Figure 16a [96]. (The PEG-PEI-dex concentration is 3 wt % and the ratio of the concentration of γ-CD to the repeating PEI-PEI unit is 1:4.) The addition of γ-CD to the PEG-PEI-dex solution causes viscosity to rise from ~10^−1^ to 10^2^ Pa·s at pH 10. Upon lowering the PEG-PEI-dex/γ-CD solution pH to 4, under which conditions all of the PEI secondary amine groups are protonated, solution viscosity decreases by three orders of magnitude consistent with a loosening of the hydrogel network in which probably only the PEG segments of PEG-PEI-dex/γ-CD are complexed by γ-CD (Figure 16b).

In contrast, when α-CD is added to a PEG-PEI-dex solution at pH 10 under the same conditions as for the addition of γ-CD, there is little change in viscosity consistent with the smaller α-CD annulus only accommodating a single PEG-PEI strand in its host–guest complex and consequently not forming a cross-link between PEG-PEI-dex chains. However, under different conditions, when several α-CD thread onto a single polymer chain to form a polyrotaxane they may aggregate in a localized crystalline state to effectively form cross-links between the polyrotaxanes in a hydrogel network [97–99].

A group of pH-responsive hydrogels which comprises four adamantyl-substituted polyacrylamides, in which the adamantyl tether varies in length, and either a linear or a globular β-CD polymer in which the β-CD are cross-linked with epichlorohydrin has been reported by Koopmans and Ritter et al. [100]. The hydrogel viscosities vary substantially with the concentrations of the two polymers and the length of the adamantyl tether. Thus, when the tether length between the adamantyl substituents and the polymer backbone increases progressively from a single amido group through -CONH(CH)_2_CONH-, to -CONH(CH)_5_CONH- to -CONH(CH)_11_CONH- the zero-shear viscosities in the presence of the linear β-CD host polymer vary in the sequence 3.63, 1007, 354.8 and 138.3 Pa·s at 293.2 K and pH 7 (when both polymer concentrations are 50 mg/L). When the tether consists only of an amido group the adamantyl substituents are too crowded by the polymer backbone to complex strongly with the β-CD substituents. An increase in tether length to -CONH(CH)_2_CONH-, maximizes the host–guest complexation and retains substantial stiffness in the hydrogel. When the tether further lengthens to -CONH(CH)_5_CONH- and -CONH(CH)_11_CONH- host–guest complexation is unlikely to be hindered, but the increase in tether length decreases the hydrogel stiffness proportionately. In the pH range 4–6, the zero-shear viscosity of the hydrogel formed from the adamantyl polymer with the -CONH(CH)_5_CONH- tether and the globular β-CD polymer shows little variation. However, zero-shear viscosity doubles at pH 10 consistent with deprotonation of the adamantyl polymer which results in an increase in its volume, as shown by the increase in the hydrodynamic diameter of the adamantyl polymer alone from 3.12 nm at pH 6 to 4.85 nm at pH 10. This allows more adamantyl substituents to be complexed by the β-CD substituents of the β-CD-substituted polymer such that the aggregation of the hydrogel network increases.

#### 5.3 Thermo-responsive materials

**Figure 17:**
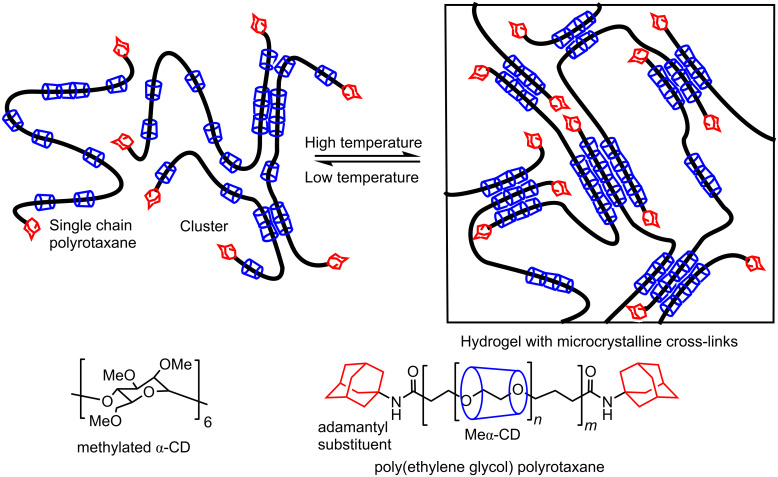
Structure of poly(ethylene glycol) polyrotaxane with adamantyl end substituents, and its temperature dependent equilibrium between the solution and hydrogel states [101].

In 2006, Kataoka et al. showed that an aqueous solution of the poly(ethylene glycol) polyrotaxane with adamantyl end-substituents (Figure 17) changes from a solution of single chains and small clusters of polyrotaxanes at low temperature to an elastic hydrogel containing microcrystalline aggregates of the methylated-α-CD components of the hydrogel at higher temperatures using differential scanning microcalorimetry, rheology, X-ray diffractometry and ^1^H NMR spectroscopy [101]. The average molecular weight of the poly(ethylene glycol) component of the polyrotaxane was 35 kDa and it was estimated that there were ≈110 methylated-α-CD threaded onto each polyrotaxane chain. At low temperatures, hydrophobic interactions among the methylated α-CD result in forming small clusters and, with increase in temperature, these clusters grow into stable crystal-like structures such that the hydrogel functions similarly to a block copolymer with hard segments composed of micro-crystalline methylated α-CD and softer segments composed of polyethylene glycol in the hydrogel.

**Figure 18:**
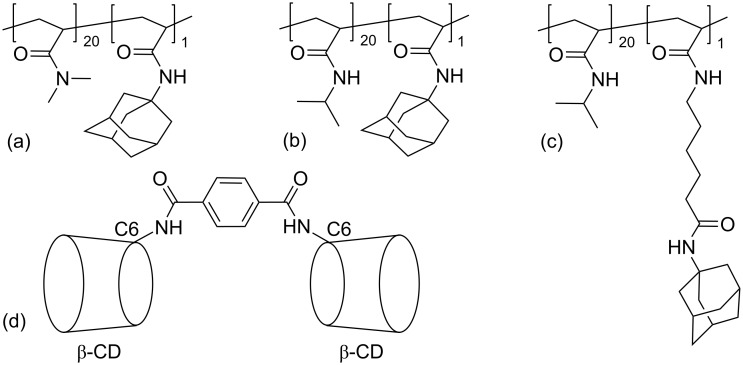
Copolymers of either (a) *N*,*N*-dimethylacrylamide (DMAA) or (b) *N*-isopropylacrylamine (NIPAAM) with 1-adamantylacrylamide, and (c) copolymer of NIPAAM with 6-acryloylaminohexanoic acid [102]. (d) The β-CD dimer in which substitution is at the C6 carbon of a D-glucopyranose subunit of each β-CD.

The preparations of the copolymers of either *N*,*N*-dimethylacrylamide (DMAA) or *N*-isopropylacrylamine (NIPAAM) with 1-adamantylacrylamide, and of the copolymer of NIPAAM with 6-acryloylaminohexanoic acid in which the ratio of the acrylamide units to adamantyl subunits is 20:1 have been reported by Ritter et al. [102]. They find that the viscosity of 50 g/L aqueous solutions of the three copolymers increases greatly within seconds after mixing with a β-CD dimer (Figure 18) to form stable hydrogels through ditopic host–guest complexation of the adamantyl substituents forming cross-links between the copolymer chains. The DMAA-based adamantyl-substituted copolymer/β-CD dimer hydrogel shows no turbity change in the range of 283.2–363.2 K probably because it is the least hydrophobic of the three polymers. In contrast, those of the two NIPAAM-based adamantyl-substituted/β-CD dimer hydrogels show temperature dependent turbidity with cloud points at 287.2 K and 288.9 K with increase in polymer backbone to adamantyl tether length. These compare with cloud points of 296.2 K and 294.2 K for the two respective NIPAAM-based adamantyl-substituted copolymers alone.

**Figure 19:**
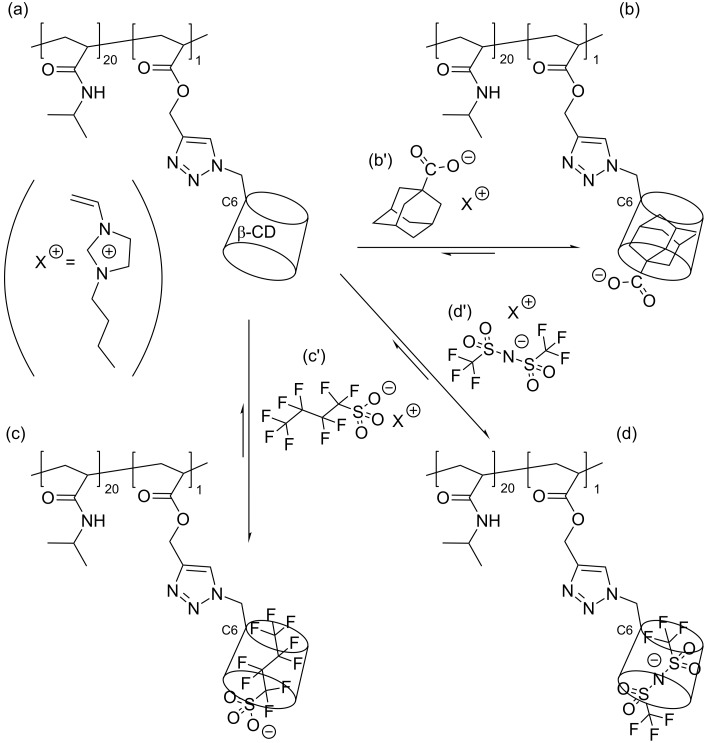
The copolymer of isopropylacrylamine and methacrylated β-CD (a) and its complexation of the anions of the ionic liquids 1-butyl-3-vinylimidazolium-adamantanecarboxylate, -bis(trifluoromethyl sulfonyl amide and -nonafluorobutansulfonate, (b’), (c’) and (d’), respectively to give the copolymer complexes (b), (c) and (d) [103].

A related study involves the copolymer of isopropylacrylamine and methacrylated β-CD (a) in Figure 19 and the complexation of the anions of the ionic liquids 1-butyl-3-vinylimidazolium-adamantanecarboxylate, -bis(trifluoromethylsulfonylamide and –nonafluorobutansulfonate, (b’), (c’) and (d’), respectively in Figure 19, by the β-CD substituent of the copolymer to form the copolymer complexes (b), (c) and (d) [103]. (β-Cyclodextrin forms 1:1 host–guest complexes with (b’), (c’) and (d’) to give complexes characterized by complexation constants 10^−3^*K*_11_ = 5.3, 21.0 and 8.1 M^−1^ in aqueous solution at 298.2 K [104].) The three copolymer complexes (b)–(d) are effectively pseudopolyanions and this has interesting behavioral consequences. Thus, turbidity measurements in aqueous solution show the cloud point for (a) to be 309.2 K whereas those of (b) and (c) are 316.2 K and 326.2 K, respectively. These increases are attributed to an increase in hydrophilicity caused by the anionic carboxylate and sulfonate groups protruding from the β-CD annuli and interacting with water. However, in (d) the negative charge is located in the centers of the β-CD annuli and there is no enhancement of interaction with water and the cloud point occurs at 307.2 K.

Light-scattering studies show the hydrodynamic diameters of (a)–(d) to be 15.1, 11.5, 9.8 and 16.5 nm in water at 298.2 K. The decrease in hydrodynamic diameter from (a) to (b) and (c) is attributable to simultaneous ion-pairing between the 1-butyl-3-vinylimidazolium cations and either the anionic carboxylate or sulfonate groups causing overall attraction between the host–guest complex substituents of (b) and (c) and a decrease in polymer coil size by comparison with (a). Conversely, the location of the negative charges in the centers of the β-CD annuli of (d) decreases ion-pairing and repulsion between the 1-butyl-3-vinylimidazolium cations slightly increases polymer coil size by comparison with (a).

#### 5.4 Redox-responsive materials

Redox-responsive hydrogel systems have potential applications as environmentally benign electro-functional materials. Such systems require a redox couple as a central component. One such system is that in which the oxidation states of iron are 0 and I in ferrocenecarboxalate, [Fe^0^(C_5_H_5_)(C_5_H_5_CO_2_^−^)]^−^ (FCA^−^) and [Fe^I^(C_5_H_5_)(C_5_H_5_CO_2_^−^)] (FCA), respectively, whose interactions with β-CD in basic aqueous solution were studied by Evans et al. in 1985 [105]. A 1:1 β-CD·FCA^−^ host–guest complex characterized by a complexation constant *K* = 2.2 × 10^3^ M^−1^ at 293.2 K forms, but β-CD·FCA has a much lower *K* ≤ 20 M^−1^. Thus, the oxidation state of iron determines the relative stabilities of β-CD·FCA^−^ and β-CD·FCA. Conjointly, these complexes may potentially be used as an electrochemical switch in a supramolecular system.

**Figure 20:**
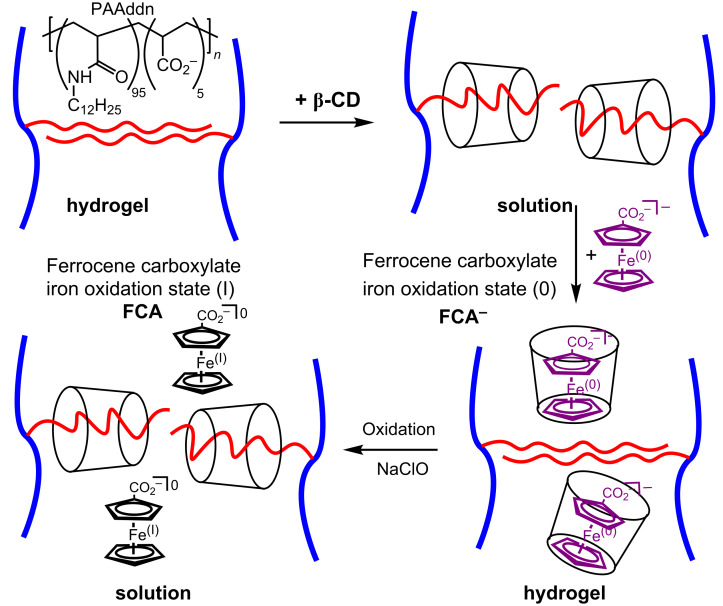
Solution to hydrogel transitions for two segments of PAAddn in the presence of β-CD and change in the ferrocenecarboxylate oxidation state in basic aqueous solution [106].

In 2006, Harada et al. realized this potential in a redox-responsive hydrogel system constructed from β-CD, PAAddn and FCA^−^ (Figure 20) [106]. The hydrophobic association between the *n*-dodecyl substituents, *n*-C_12_H_25_, produces cross-links between PAAddn chains and the formation of a PAAddn hydrogel. Addition of β-CD results in a strong complexation of the dodecyl substituents and a free flowing solution. Subsequent addition of FCA^−^ (Fe(0)) results in preferential complexation between β-CD and FCA^−^ such that the PAAddn hydrogel reforms. This situation is reversed upon oxidation of FCA^−^ with sodium hypochlorite to FCA (Fe(I)) which is complexed much less strongly by β-CD than are the dodecyl substituents of PAAddn.

An interesting variation on the redox chemistry of ferrocene in polymer systems was presented by Zhu et al. who attached ferrocene, FC, as a substituent to branched poly(ethylene imine), BPEI, through reaction with ferrocenecarboxaldehyde to give the ferrocene substituted polymer, BPEI-FC [107]. Aqueous solutions of this polymer are about ten times more viscous than the precursor BPEI polymer as a consequence of the enhancement of polymer chain association because of the hydrophobicity of the ferrocene substituents of BPEI-FC. However, this viscosity is greatly deceased upon the addition of β-CD because host–guest complexation of ferrocene masks its hydrophobicity and the hydrophilic exterior of the complexing β-CD much decreases association between the polymer chains. The same effect occurs when hydrogen peroxide is added to aqueous BPEI-FC and the ferrocene iron(0) is oxidized to ferrocene iron(I). With iron in oxidation state I, the ferrocene substituents assume uni-positive charges and consequently aggregate weakly with a corresponding decrease in solution viscosity.

#### 5.5 Self-healing systems

**Figure 21:**
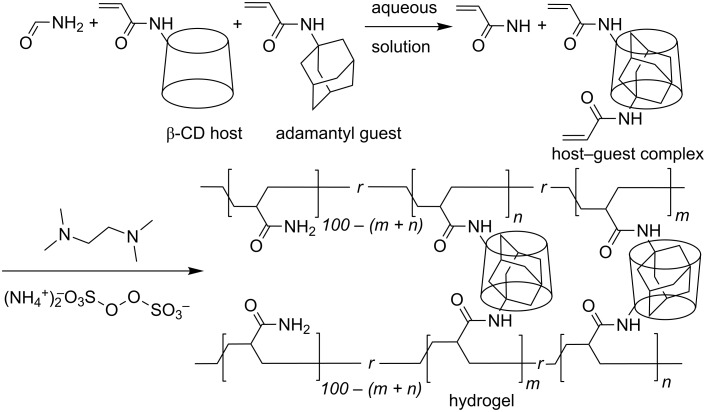
Preparation of a β-CD and adamantyl substituted acrylamide polymer hydrogel involving host–guest complexation between their β-CD and adamantyl substituents [108].

Because of their ability to form host–guest complexes in water, cyclodextrins have attracted attention as components of self-healing materials. Thus, Harada et al. constructed self-healing supramolecular hydrogels from poly(acrylamide) substituted with both cyclodextrins and aliphatic substituents. This is exemplified by one such system in which the radical copolymerization of aqueous acrylamide, acrylamide substituted β-CD and *N*-adamantyl-acrylamide gives a β-CD- and adamantyl-substituted poly(acrylamide) which forms a hydrogel as shown in Figure 21 [108]. When a portion of the hydrogel is cut in two and both halves are brought back into close contact, the cut rapidly self-heals as β-CD/adamantyl host–guest complexation re-establishes inter-polymer chain links between the two halves. A similar situation occurs with the analogous polymer in which β-CD and the adamantyl substituents in Figure 21 are replaced by α-CD and *n*-butyl substituents, respectively.

**Figure 22:**
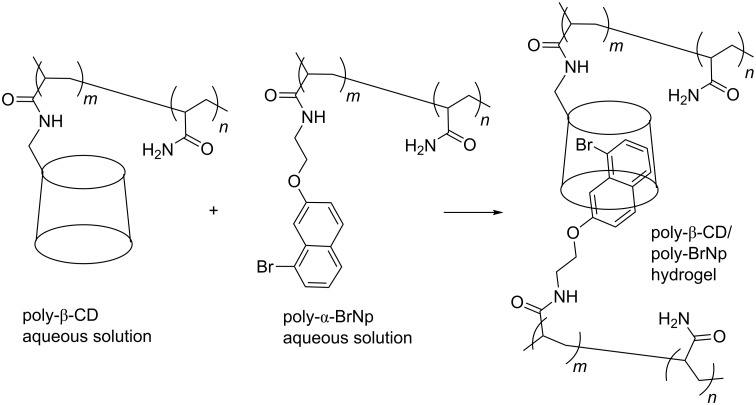
Aqueous solutions of the polymers poly-β-CD and poly-α-BrNP form the poly-β-CD/poly-α-BrNP hydrogel [109].

Subsequently, Tian et al. reported the formation of a self-healing polymeric hydrogel based on the host–guest interaction between the β-CD substituents of an acrylamide-based polymer, poly-β-CD, and the α-bromonaphthalene substituents of a second acrylamide-based polymer, poly-α-BrNp (Figure 22) [109]. A hydrogel forms rapidly when aqueous solutions of poly-β-CD and poly-α-BrNP are mixed. When a solid sample of this hydrogel is cut in two, it rapidly self-heals within a minute through reforming host–guest complexes between the β-CD and α-bromonaphthalene substituents of poly-β-CD and poly-α-BrNP. Another interesting aspect is that because the α-bromonaphthalene substituents occupy the hydrophobic β-CD annuli in the hydrogel, UV radiation induces room temperature phosphorescence which, in combination with the self-healing properties of the hydrogel, may lead to some interesting applications.

**Figure 23:**
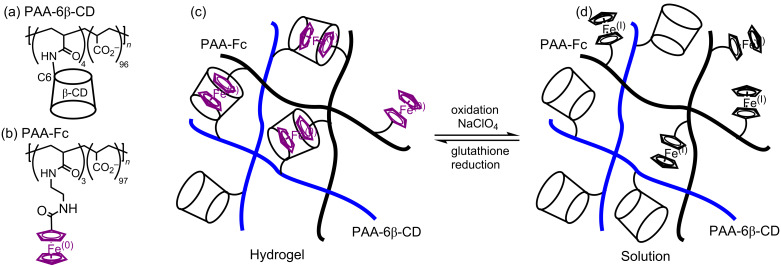
(a) Randomly β-CD substituted poly(acrylate), PAA-6β-CD. (b) Randomly ferrocenyl substituted poly(acrylate), PAA-Fc. (c) PAA-6β-CD/PAA-Fc hydrogel. (d) PAA-6β-CD/PAA-Fc solution after ferrocenyl iron(0) oxidation to ferrocenium iron(I) [110].

The change of oxidation state of polymer substituent ferrocene iron(0) to more hydrophilic ferrocenium iron(I) can also result in interesting self-healing characteristics as is the case for the hydrogel formed between randomly β-CD substituted poly(acrylate), PAA-6β-CD and randomly ferrocenyl substituted poly(acrylate), PAA-Fc shown as (a) and (b), respectively in Figure 23 [110]. Thus, in aqueous solution the β-CD substituents of PAA-6β-CD complex the ferrocenyl substituents of PAA-Fc to form the hydrogel (c) which reverts to a solution of polymer chains when the hydrophobic ferrocenyl substituents of PAA-Fc are oxidized by sodium perchlorate to hydrophilic ferrocenium substituents. This oxidation may be reversed with glutathione to reform the hydrogel. At the macroscopic level a hydrogel cube may be cut in halves which when pressed together re-establish host–guest complexation of the ferrocenyl substituents by the β-CD substituents to self-heal. This self-healing may be controlled by addition of sodium perchlorate solution to the cut surface, whereby oxidation of the ferrocenyl substituent prevents self-healing. Subsequent addition of glutathione solution to the same surface reverses this situation and the self-healing properties are restored.

**Figure 24:**
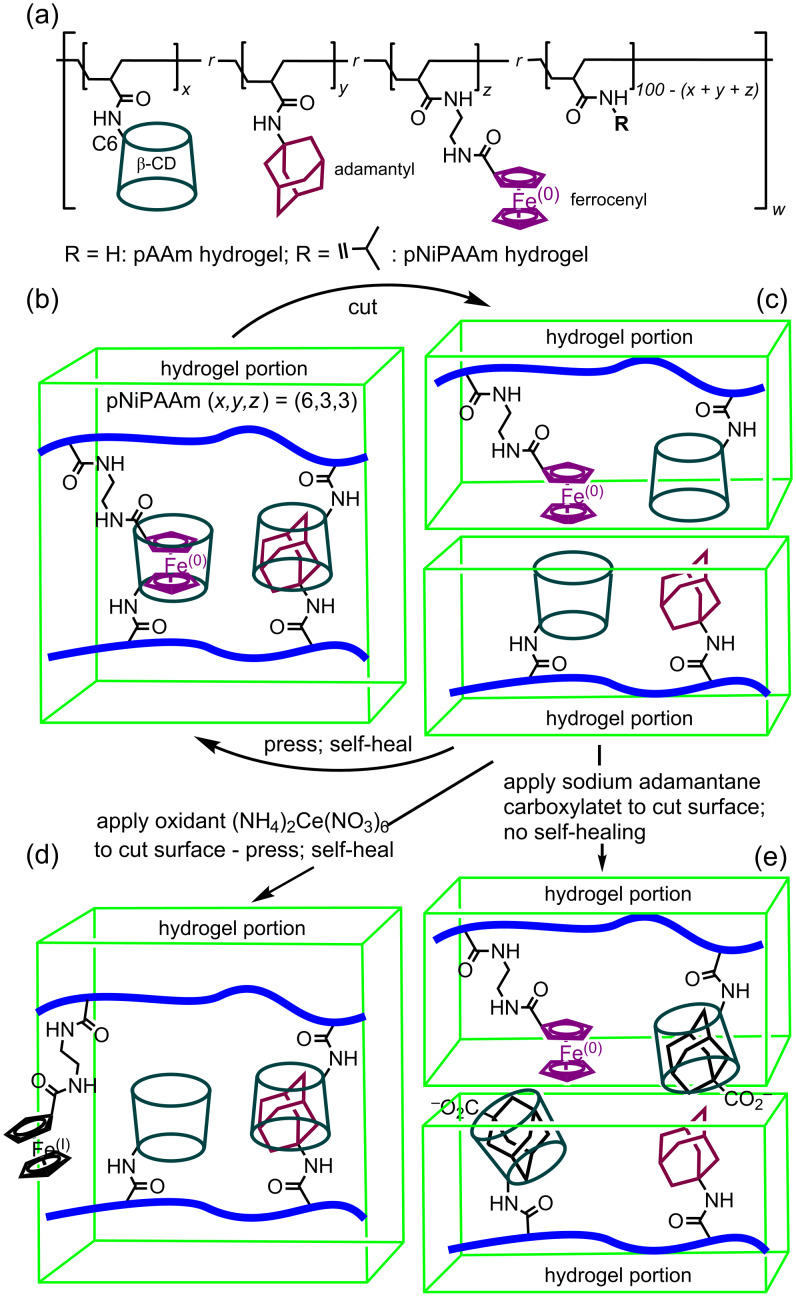
(a) The β-CD, adamantyl and ferrocenyl substituted pAAm and pNiPAAM polymers. (b) The β-CD, adamantyl and ferrocenyl substituted pNiPAAM hydrogel where the substituent mol % ratio *x*:*y*:*z* is 6:3:3. (c) Cutting of a hydrogel cube and self-healing of the two halves after pressing together. (d) Oxidation of the ferrocenyl (Fe(0)) substituent to positively charged ferrocenium (Fe(I)) through oxidation with (NH_4_)_2_Ce(NO_3_)_6_ on the surfaces of the halves followed by pressing and self-healing. (e) Addition of sodium adamantane carboxylate to the surfaces of the halves results in competitive host–guest complexation and an absence of self-healing [111].

The simultaneous substitution of a host and two different guest substituents onto a single polymer presents opportunities for variations in self-healing properties to be incorporated as has been explored by Harada et al. with the β-CD, adamantyl and ferrocenyl substituted poly(acrylamide) (pAAm) and poly(*N*-isopropylacrylamide) (pNiPAAM) hydrogels shown in Figure 24 [111]. Thus, a cube of (pNiPAAM) where the mol % ratio of β-CD to adamantyl to ferrocenyl substituents is 6:3:3 may be cut into halves and upon pressing the halves together self-healing occurs through host–guest complexation ((b) and (c)). However, if one of the cut surfaces is treated with (NH_4_)_2_Ce(NO_3_)_6_ oxidation of iron(0) in the ferrocenyl substituent to iron(I) renders the resulting ferrocenium substituent hydrophilic such that it does not complex in the β-CD substituent annulus ((c) and (d)). Nevertheless, upon pressing the two halves together self-healing still occurs through β-CD substituent/adamantyl substituent complexation. Finally, if adamantane carboxylate is applied to one of the cut surfaces in sufficient quantity and the two halves are pressed together, competitive β-CD substituent/adamantane carboxylate complexation prevents self-healing ((c) and (e)). The properties of this hydrogel can also be utilized in controlling expansion and contraction and shape memory. The practical applications which potentially flow at the macroscopic level from such host–guest chemistry are substantial [112–114].

## Conclusion and Perspective

In this brief review it is shown that there is a plethora of routes to supramolecular polymer networks in aqueous solution based on cyclodextrin host–guest complexation. Through variations in this supramolecular chemistry at the molecular level macroscopic properties may be tailored to give smart-materials possessing stimuli responsive characteristics exemplified by photo-, pH-, thermo-, and redox-responsivity and self-healing. Inevitably, many more novel polymer network systems incorporating cyclodextrins will appear; some of which will find exciting applications.
